# Prevalence of *Dirofilaria immitis* among shelter dogs in Tokyo, Japan, after a decade: comparison of 1999–2001 and 2009–2011

**DOI:** 10.1051/parasite/2014008

**Published:** 2014-03-03

**Authors:** Masaaki Oi, Souichi Yoshikawa, Yasuaki Ichikawa, Kazuhide Nakagaki, Jun Matsumoto, Sadao Nogami

**Affiliations:** 1 Laboratory of Medical Zoology, Department of Veterinary Medicine, College of Bioresource Sciences, Nihon University, 1866 Kameino Fujisawa Kanagawa 252-0880 Japan; 2 Tokyo Metropolitan Institute of Public Health, 3–24–1 Hyakunincho Shinjuku Tokyo 169-0073 Japan; 3 Merial Japan Limited, 3–20–2 Nishi Shinjyuku Shinjyuku Tokyo 163-1488 Japan; 4 Laboratory of Infectious Diseases and Immunology, College of Veterinary Medicine, Nippon Veterinary and Life Science University, 1–7–1 Kyonancho Musashino Tokyo 180-8602 Japan

**Keywords:** Canine heartworm disease, Chemoprophylaxis, *Dirofilaria immitis*, Dog, Epidemiology

## Abstract

Changes in the seroprevalence of *Dirofilaria immitis* infection among shelter dogs between a decade ago and the present were evaluated. Serum samples were collected from 200 adult dogs in urban and suburban areas in Tokyo, Japan, during two 2-year periods (April 1999 to March 2001 and April 2009 to March 2011). Sera were tested for the presence of *D. immitis* antigen using a specific commercialized kit. The seroprevalence of *D. immitis* infection was 46% in 1999–2001 and 23% in 2009–2011. A decrease was observed in the prevalence of infection between 1999–2001 and 2009–2011; in particular, the prevalence in urban areas decreased significantly compared with that in suburban areas (*P* < 0.01). There was no significant difference in prevalence between the sexes in each period, but there was a significant difference between mixed-breed and purebred dogs (*P* < 0.01). The decrease in prevalence of canine heartworm disease in urban areas could be related to better veterinary care.

## Abstract (fr)

Les changements de séroprévalence d’infection à *Dirofilaria immitis* parmi les chiens en chenil ont été évaluées il y a une décennie et récemment. Des sérums provenant de 200 chiens adultes ont été collectés dans des zones urbaines ou péri-urbaines de Tokyo pendant deux périodes de deux ans (Avril 1999–Mars 2001 et Avril 2009–Mars 2011). Les sérums ont été testés pour la présence d’antigènes de *D. immitis*. La séroprévalence de l’infection par *D. immitis* était de 46 % en 1999–2001 et 23 % sur la période 2009–2011. Une diminution de la prévalence a été observée entre 1999–2001 et 2009–2011. En particulier, la prévalence en zones urbaines a significativement diminué par rapport à celle des zones péri-urbaines (*P* < 0.01). Aucune différence significative n’a été notée entre les sexes pour chaque période mais il y avait une différence significative entre les chiens de race et les autres (*P* < 0.01). La diminution de prévalence de la dirofilariose canine observée en zone urbaine pourrait être liée à un meilleur suivi vétérinaire.

## Introduction

Cardiopulmonary dirofilariosis is a parasitic disease with a worldwide distribution caused by *Dirofilaria immitis* Leidy, 1856 [9], a nematode that is transmitted by mosquitoes. The adult worms are commonly found in the pulmonary arteries and right ventricle of dogs, and result in pathological damage, such as edema, ascites, arrhythmia, tachyarrhythmia, and even death of infected dogs [10]. The increasing temperatures caused by climate change and global warming can affect the distribution and density of mosquitoes, and consequently the transmission and spread of mosquito-borne diseases, such as dirofilariosis caused by *D. immitis* [3, 5]. Predictive models in Europe and Argentina have demonstrated an actual risk of spread of *Dirofilaria* spp. infections into areas previously free from the disease [4, 5, 11, 14].

In Japan, *D. immitis* infection of dogs has been considered to be prevalent since the earliest report on canine dirofilariosis in 1880 [1]. In the latest study, Nogami and Sato [12] reported a prevalence rate of 46.8% in shelter dogs tested at necropsy from 1989 to 1995. However, current epidemiological information on the prevalence of *D. immitis* infection is limited. The present study was conducted to determine the prevalence of *D. immitis* infection among shelter dogs in Tokyo, Japan, in order to evaluate the modifications in the prevalence between the present and a decade ago, and between urban and suburban areas.

## Materials and methods

**Figure 1.**
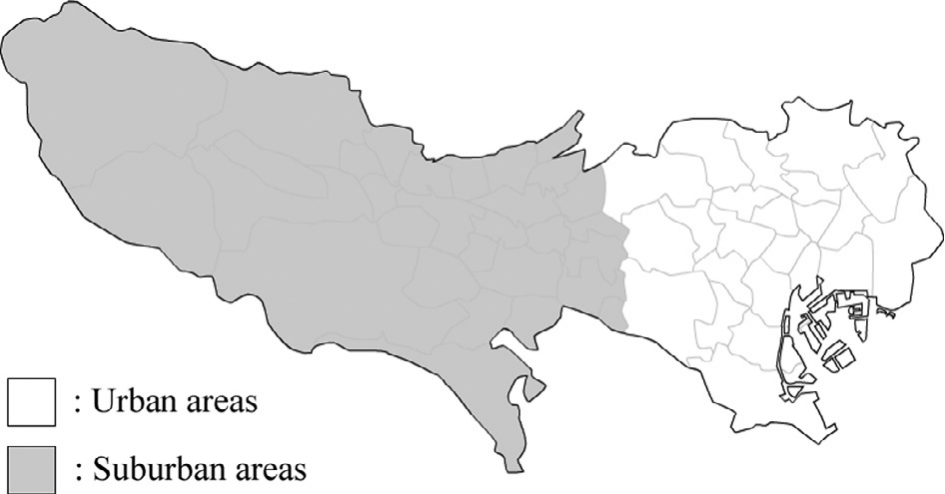
Urban and suburban areas: regions of origin of the shelter dogs tested for *Dirofilaria immtis* infection.

The present study was carried out in Tokyo, Japan (Figure 1). Canine serum samples were obtained from 200 adult shelter dogs kept in the Tokyo Metropolitan Animal Care and Consultation Center (Permission No. 22-2350). All sampling procedures were performed by shelter veterinarians in accordance with the Guidelines on research and survey using animals at Tokyo Metropolitan Animal Care and Consultation Center. This included samples from 100 dogs from April 1999 to March 2001, and 100 dogs from April 2009 to March 2011. A questionnaire including sex, estimated age, breed, and housing area (urban or suburban) was collected for each dog. Serum was stored at −30 °C until assay. All samples were tested for circulating *D. immitis* antigen using a specific commercialized immunochromatography kit (Solo Step^®^ CH; Heska Co., Loveland, CO, USA) according to the manufacturer’s instructions.

The data were analyzed to evaluate the differences between proportions of dogs infected with *D. immitis* using the chi-square test. In all analyses, *P* < 0.05 was taken to indicate statistical significance.

## Results and discussion

The research area, sex and breed of distribution in dogs with *D. immitis* infection are shown in Table 1. The overall prevalence rates of *D. immitis* infection were 46.0% in 1999–2001 and 23.0% in 2009–2011 (*P* < 0.01). The prevalence rates of infection in urban areas were 46.0% in 1999–2001 and 18.2% in 2009–2011 (*P* < 0.01), and those in suburban areas were 46.0% and 28.9% (*P* > 0.05), respectively. There was no significant difference in prevalence between male and female dogs between the periods. The prevalence rates in mixed-breed dogs were 58.5% in 1999–2001 and 50.0% in 2009–2011, while those in purebred dogs were 22.9% and 10.3%, respectively. Significant differences (*P* < 0.01) in prevalence were observed between mixed-breed and purebred dogs in both periods.

**Table 1.** Seroprevalence of *Dirofilaria immitis* infection in shelter dogs in April 1999 to March 2001 and April 2009 to March 2011 in Tokyo, Japan.

Dogs	Prevalence	
	1999–2001	2009–2011
Research area		
Urban areas	46.0% (23/50)^a^	18.2% (10/55)^a^
Suburban areas	46.0% (23/50)	28.9% (13/45)
Sex		
Male	46.0% (23/50)^b^	24.0% (12/50)^b^
Female	46.0% (23/50)^c^	22.0% (11/50)^c^
Breed		
Mixed breeds	58.5% (38/65)^d^	50.0% (16/32)^e^
Purebreds	22.9% (8/35)^d^	10.3% (7/68)^e^
Total	46.0% (46/100)^f^	23.0% (23/100)^f^

a,d,e,f The different letters indicate a significant difference among groups by chi-square test (*P* < 0.01).

b,c The different letters indicate a significant difference among groups by chi-square test (*P* < 0.05).

In the present study, a significant decrease from 46.0% to 23.0% was observed in the overall prevalence of *D. immitis* infection in Tokyo over the past decade. During the past decade, various types of macrocyclic lactones for *D. immitis* prevention have come into use worldwide, and the increased use of chemoprophylaxis is probably the major factor responsible for the decrease in prevalence of *D. immitis* infection in Tokyo. However, there are contrasting results in terms of breeds. The prevalence of infection in purebreds decreased by half over the past decade (22.9% in 1999–2001 and 10.3% in 2009–2011), while the infection in mixed breeds was still highly prevalent (58.5% in 1999–2001 and 50.0% in 2009–2011). This could reflect a higher rate of chemoprophylaxis in the purebred canine population than that in the mixed-breed canine population in Tokyo.

Decreases in prevalence of *D. immitis* infection were observed in both urban and suburban areas. These findings suggest that the prevalence of infection is markedly affected by veterinary care. The decreased prevalence, particularly in urban areas, has been conspicuous compared with that in suburban areas. This is because the rate of purebred populations in the urban areas increased and most of these dogs probably received chemoprophylaxis. Alternatively, urban areas with less parks may be more susceptible to changes in the environment than suburban areas.

The results of the present study were compared with those of other studies performed in different countries on approximately the same latitude as Japan. The prevalence of *D. immitis* infection in dogs in these countries varied markedly: 6.9%–20.9% in Korea [7, 13], 0.33%–3.33% in China [15], 3.6%–8.9% in Portugal [2], 34.13% in Greece [6], and 1%–12.5% in the United States [8]. The present study demonstrated that *D. immitis* infection is endemic in Japan. The differences in prevalence among countries may be due to the differences in density of vectors, density of the parasite reservoir, and rate of prevention in dogs. This is the first study to evaluate changes in the seroprevalence of *D. immitis* infection in a metropolitan city over the past decade.

## Conflict of interest

The authors declare that there are no conflicts of interest.
